# Construction of consolidated bio-saccharification biocatalyst and process optimization for highly efficient lignocellulose solubilization

**DOI:** 10.1186/s13068-019-1374-2

**Published:** 2019-02-18

**Authors:** Shiyue Liu, Ya-Jun Liu, Yingang Feng, Bin Li, Qiu Cui

**Affiliations:** 1 CAS Key Laboratory of Biofuels, Shandong Provincial Key Laboratory of Synthetic Biology, Qingdao Institute of Bioenergy and Bioprocess Technology, Chinese Academy of Sciences, Qingdao, China; 2 Dalian National Laboratory for Clean Energy, Chinese Academy of Sciences, Dalian, China; 3 University of Chinese Academy of Sciences, Chinese Academy of Sciences, Beijing, China

**Keywords:** Cellulase, Cellulosome, *Clostridium thermocellum*, Fermentable sugar, Lignocellulose, Saccharification

## Abstract

**Background:**

The industrial conversion of biomass to high-value biofuels and biochemical is mainly restricted by lignocellulose solubilization. Consolidated bio-saccharification (CBS) is considered a promising process for lignocellulose solubilization depending on whole-cell biocatalysts that simultaneously perform effective cellulase production and hydrolysis. However, it usually takes a long time to reach a high saccharification level using the current CBS biocatalyst and process.

**Results:**

To promote the saccharification efficiency and reduce the cost, a *Clostridium thermocellum* recombinant strain *∆pyrF*::KBm was constructed as a new CBS biocatalyst in this study. The key CBS factors, including the medium, inoculum size and cultivation, and substrate load, were investigated and optimized. The saccharification process was also stimulated by adding free hemicellulases, suggesting the need to further enhance hemicellulase activity of the whole-cell catalyst. Under the optimal conditions, the CBS process was shortened by 50% with pretreated wheat straw as the substrate. The sugar yield reached 0.795 g/g and the saccharification level was 89.3%.

**Conclusions:**

This work provided a new biocatalyst and an optimized process of CBS and confirmed that CBS is a feasible strategy for cost-efficient solubilization of lignocellulose, which will greatly promote the industrial utilization of lignocellulosic biomass.

**Electronic supplementary material:**

The online version of this article (10.1186/s13068-019-1374-2) contains supplementary material, which is available to authorized users.

## Background

Lignocellulose is the most abundant sustainable carbon source. The conversion of lignocellulosic biomass, especially those considered to be agricultural wastes, is of worldwide importance in terms of both environmental protection and substitution of fossil sources [1–3]. Although lignocellulose is a valuable feedstock, it is not easy to use because of its complex and recalcitrant structure. Lignocellulose is mainly composed of cellulose, hemicellulose, and lignin. Cellulose is the most abundant polymer with disaccharide cellobiose as the repeating unit. Through extensive intramolecular and intermolecular hydrogen bonding networks, the glucose units are tightly bound together to form crystalline structures. Hemicellulose and lignin interact with cellulose fibers and each other to form complex linking networks and recalcitrant structures [4, 5]. Thus, the main obstacle of lignocellulose bioconversion is the deconstruction of the natural defense mechanism of plants and the cost-efficient solubilization.

Currently, three strategies are reported for lignocellulose bioconversion: separate enzymatic hydrolysis and fermentation (SHF), simultaneous saccharification and fermentation (SSF) and consolidated bioprocessing (CBP) [6]. For SHF, hydrolysis and fermentation are separately performed. SSF combines hydrolysis with fermentation to relieve the inhibitory effect of intermediate hydrolysates on the cellulases. In this way, cellulases are usually produced by fungi aerobically in a different reactor and the enzyme cost is an essential issue to consider. CBP further integrates the enzyme production, cellulose hydrolysis, and fermentation in one step to reduce cellulase and investment costs [7]. CBP requires a biocatalyst that can simultaneously hydrolyze the lignocellulose to sugars and convert sugars to target products [8].

Various biocatalysts have been constructed for lignocellulose conversion in the frame of CBP strategy. Alcohol-producing yeasts were used for producing cellulosic ethanol via CBP by expressing secretive free cellulases [9] or displaying cellulases on the cell surface [10, 11]. Co-culture methods were also developed to enhance the ethanol titer and cellulose conversion rate [12–14]. The pretreatment of lignocellulosic biomass is usually non-negligible, but an engineered extreme thermophile *Caldicellulosiruptor bescii* was reported to use unprocessed biomass, which was considered the second generation of CBP technique [15, 16]. Cellulolytic clostridia naturally solubilizing cellulosic substrates were used for CBP production of ethanol [17] and other higher alcohols [18–20]. *Clostridium thermocellum* is one of the most attractive candidates for efficient cellulose solubilization because it produces the cellulosome, a highly organized multiprotein supermolecular complex containing both enzymatic subunits and non-catalyzing scaffoldins [21]. Using the cellulosome, *C. thermocellum* has been determined a robust and effective biocatalyst that outperforms commercial fungal cellulase cocktails in lignocellulose solubilization [22–25]. However, industrial-scale application of CBP remains challenging so far because of the low lignocellulose saccharification efficiency [26]. Additionally, the product structure of CBP is relatively simple due to the limited production potential of the biocatalyst.

Consolidated bio-saccharification (CBS) is a CBP-derived strategy that separates fermentation from the integrated CBP process but only combines cellulase production and hydrolysis in one step. In this way, the CBS process can be carried out under optimal conditions, and the compromise of the hydrolysis and fermentation conditions can be avoided for high conversion efficiency. Because CBS ends at the production of fermentable sugars, it can couple various downstream fermentation processes to produce different biochemicals, including bioethanol [27]. Thus, CBS may have broad applications. However, CBS usually takes a long time to get high sugar yield and saccharification level [28]. Additionally, although CBS employs cellulolytic biocatalysts for lignocellulose solubilization and requires no additional supplementation of cellulases, the cost for the biocatalyst cultivation is still a challenging issue. In this study, we investigated and optimized the CBS parameters, including biocatalyst, medium composition, inoculum size, and cultivation to obtain a cost-effective strategy for lignocellulose solubilization.

## Results and discussion

### Construction of a novel CBS biocatalyst by genetic engineering of *C. thermocellum*

Because the cellobiose inhibition to the cellulosome is considered one of the major problems that hinder the application of *C. thermocellum* as a CBS biocatalyst, and the introduction of beta-glucosidases (BGL) can greatly release the inhibition effect, we have previously constructed a *C. thermocellum* recombinant strain *∆pyrF*::*Ca*BglA to produce a fusion protein of the cellobiohydrolase Cel48S with a heterologous BGL [28]. Although *∆pyrF*::*Ca*BglA showed high cellulosic sugar productivity, the fusion protein was expressed at a significantly decreased level compared to that of the wild-type Cel48S in the parent strain [28]. Cel48S plays key roles in the cellulosome of *C. thermocellum* for cellulose degradation [29–31]. Hence, the low expression of Cel48S may influence the saccharification efficiency.

To avoid the reduced Cel48S expression, we determined to fuse the BGL with another cellobiohydrolase Cel9K in *C. thermocellum* [32] and constructed a new *C. thermocellum* strain ∆*pyrF*::KBm using previously developed seamless genome editing system [28]. The strain ∆*pyrF*::KBm would produce a fused protein of Cel9K with BGL containing three functional modules (GH9–*Ca*BglAm–DocI) under the control of the endogenous Cel9K promoter. The fused sequence was confirmed by PCR and sequencing of the genomic DNA (Additional file 1).

**Fig. 1:**
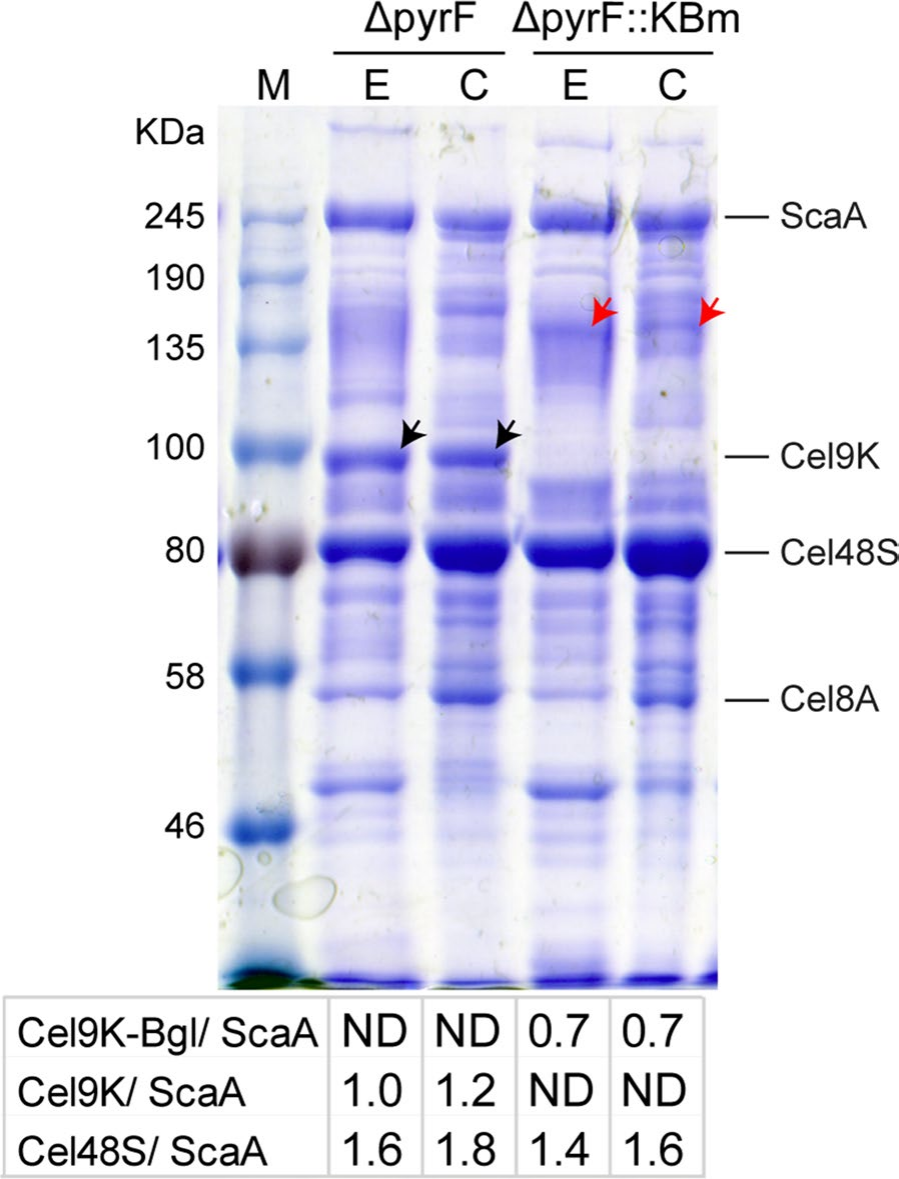
SDS-PAGE analysis of extracellular proteins (E) and cellulosomal (C) of *C. thermocellum* strains. The parent strain *∆pyrF* produced both intact Cel48S and Cel9K proteins (black arrow). ∆*pyrF*::KBm also produced the wild-type Cel48S protein as *∆pyrF*, but the Cel9K protein was rarely observed. An additional 150-kDa band was detected referring to the fusion protein Cel9K–BGL (red arrow). The intensities of the bands referring to Cel48S, Cel9K, and Cel9K–BGL were determined using the Quantity One software and were divided by that of the corresponding ScaA protein as previously described [33]. The obtained ratios shown below the lanes stand for the expression levels of the proteins. ND, not detected. Bands corresponding to known cellulosomal proteins are identified to the right of the Coomassie blue-stained gel. M, protein standards

The cellulosomal and extracellular proteins of ∆*pyrF*::KBm were prepared and analyzed by SDS-PAGE to confirm the expression of the fusion protein with a theoretical size of ~ 150 kDa. The samples from the parent strain Δ*pyrF* and the previously constructed strain Δ*pyrF*::*Ca*BglAm were also analyzed in parallel. Compared to the parent strain Δ*pyrF*, an additional 150-kDa band was detected for ∆*pyrF*::KBm but the ~ 90-kDa band referring to the wild-type Cel9K protein was rarely observed. This indicated that ∆*pyrF*::KBm produced the fusion protein instead of the Cel9K protein (Fig. 1). The intensities of the bands referring to the primary scaffoldin ScaA, Cel48S, Cel9K and the fusion protein Cel9K–BGL were determined using the Quantity One software based on the peak intensity analysis, and the relative protein expression levels were determined by dividing their band intensities with that of the ScaA protein according to a previously described ScaA-based estimation method [33]. The Cel48S protein was with comparative expression level in Δ*pyrF* and ∆*pyrF*::KBm, indicating that the expression of Cel48S was not influenced in ∆*pyrF*::KBm. However, reduced expression of Cel9K–Bgl was detected in ∆*pyrF*::KBm compared to Cel9K in Δ*pyrF*. In consideration of various molecular weights (~ 100 kDa for Cel9K and ~ 150 kDa for Cel9K–BGL), the expression intensity of Cel9K–BGL was only about 40% of that of Cel9K in Δ*pyrF* (Fig. 1). Enzyme assay showed the cellulosomes of ∆*pyrF*::KBm and Δ*pyrF*::*Ca*BglAm had the BGL activity of 13.1 ± 0.3 and 14.7 ± 0.2 U/mg, respectively, indicating ∆*pyrF*::KBm could express cellulosomal BGL at a comparative level of the previously constructed biocatalyst.

### The biocatalyst ∆*pyrF*::KBm showed higher cellulose saccharification efficiency

The strain *∆pyrF*::KBm was then used as a whole-cell biocatalyst for CBS using 100 g/L Avicel or 40 g/L sulfite-pretreated wheat straw substrate (SPS) as the sole carbon source with the inoculum size of 100% or 1%, respectively. SPS contains 65.31% of cellulose, 16.26% of hemicellulose and 9.92% of lignin. The parent strain *∆pyrF* and the previously constructed biocatalyst Δ*pyrF*::*Ca*BglAm were also used for saccharification under the same conditions.

**Fig. 2:**
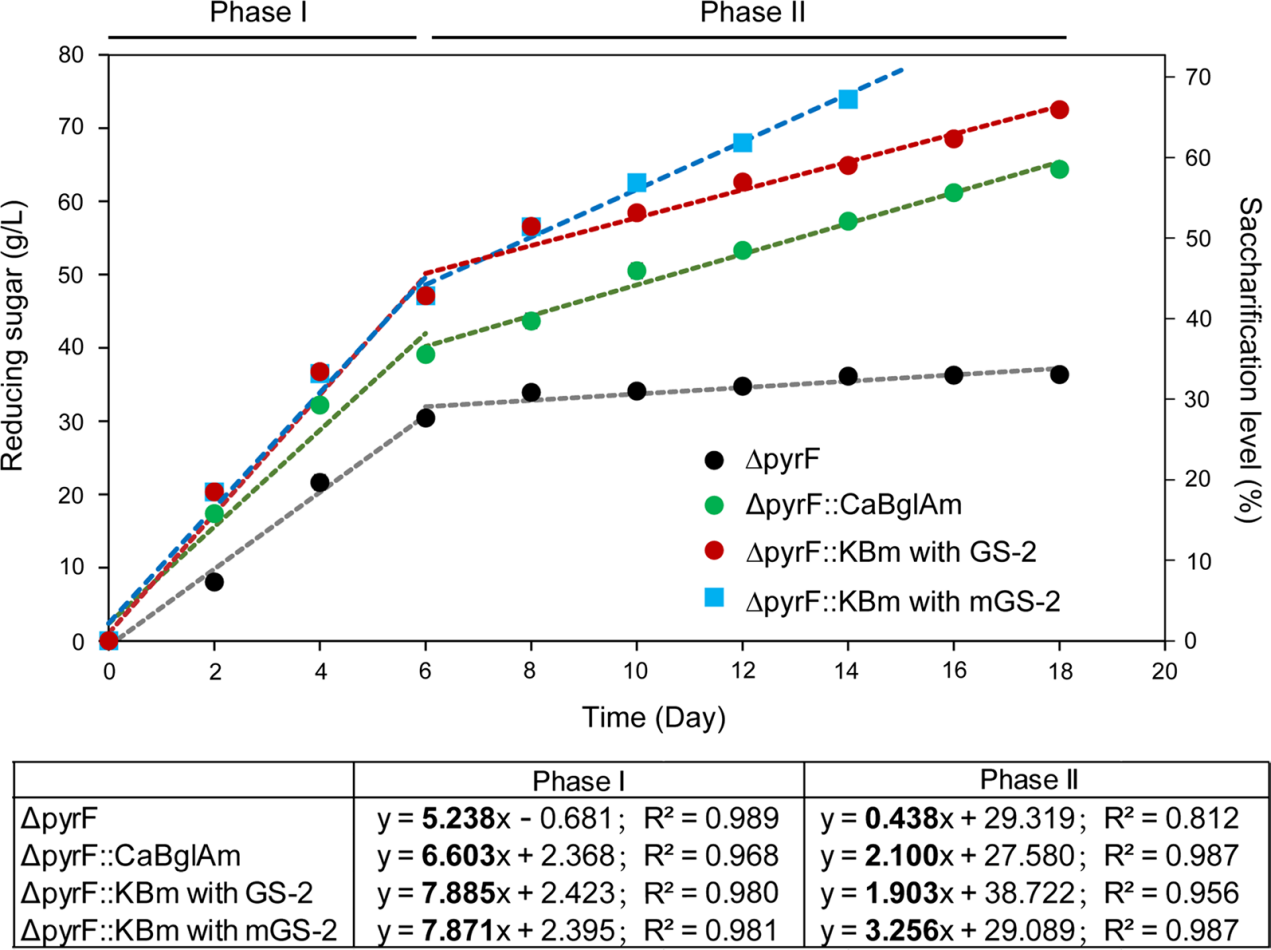
Cellulose saccharification of *C. thermocellum* strains with 100 g/L Avicel as the substrate. The Avicel saccharification of Δ*pyrF*::*Ca*BglAm and Δ*pyrF* was performed in GS-2 medium. Both GS-2 and the modified medium mGS-2 were used for the saccharification of *∆pyrF*::KBm. The inoculum size was 10%. The saccharification level was calculated by dividing the initial cellulose in the substrate (~ 110 g/L in glucose equivalence) with the amount of the obtained reducing sugar, which was determined by the DNS method. The saccharification process could be divided into two phases before and after the 6th day. The linear trend lines were calculated for both Phase I and II, and the slope (in bold) and *R*^2^ values are shown in the table. The slope values of the trend lines stand for the sugar production rates (g/L/day). Three independent experiments were performed for every strain to calculate the average values and standard errors

With 100 g/L of Avicel as the cellulosic substrate, *∆pyrF*::KBm produced 72.5 g/L reducing sugar in 18 days and the saccharification level was 65.9%, which is higher than that of *∆pyrF* or Δ*pyrF*::*Ca*BglAm (33% and 58.5%, respectively). The saccharification process of the *C. thermocellum* strains showed two phases, Phase I and Phase II, including the first 6 and later days, respectively (Fig. 2), with different sugar production rates. The sugar production curves in both Phase I and II fitted to a linear relationship with *R*^*2*^ values of 0.812–0.989. The slopes of the trend lines were then calculated to determine the sugar production rates in different phases. For all tested strains, the production rates in Phase I were generally higher than those in Phase II. The reduced saccharification efficiency indicated the reduced activity of the cellulosome, which might be caused by feedback inhibition or enzymatic instability. It has been reported that the cellulosomal activity was not inhibited by the produced acids and alcohols [34], and low amount of cellooligosaccharides and cellobiose also have slight influence on the cellulosomal activity [28, 35, 36]. Thus, the decreased cellulolytic activity of the cellulosome might result from the instability of the cellulosome after the long-time reaction. The production rate of *∆pyrF*::KBm in Phase I was 7.87 g/L/day, which was 1.2- and 1.5-fold of that of Δ*pyrF*::*Ca*BglAm and Δ*pyrF*, respectively. In Phase II, *∆pyrF*::KBm and Δ*pyrF*::*Ca*BglAm had similar sugar production rates of 1.9 and 2.1 g/L/day, respectively, but the parent strain Δ*pyrF* produced a low amount of reducing sugar in this phase. This result indicated that *∆pyrF*::KBm had increased saccharification efficiency in terms of both saccharification level and sugar production rate compared to the parent strain and the previous generation biocatalyst.

In our previous study, the supplementation of free BGL protein slightly stimulated the saccharification efficiency of ∆*pyrF*::*Ca*BglA which produces a fused protein of Cel48S and *Ca*BglA [28]. 15 and 50 U/g cellulose of *Ca*BglA were supplemented in the saccharification system of ∆*pyrF*::KBm with 100 g/L Avicel as the substrate (Additional file 2). The result showed that the saccharification level was greatly stimulated to over 75% by adding 50 U/g cellulose of the purified BGL protein. Because ∆*pyrF*::KBm produced a high amount of Cel48S protein but a low amount of the fusion protein Cel9K–BGL, this result indicated the importance of matching the expression level of BGL with that of Cel48S. Thus, we consider that the key to an effective CBS biocatalyst is the high and balanced expression levels and activities of Cel48S and BGL. We have previously tried to express a dockerin-bearing BGL under the control of *cel48S* promoter using a replicating plasmid in *C. thermocellum* DSM1313 to avoid the decreased expression of cellulosomal components, but detected low BGL activity and abundance in the cellulosome [28]. It might be caused by the improper promoters or plasmid backbone. The chromosomal integration of the BGL-encoding gene could also be tried in future to obtain high expression of the free BGL.

**Fig. 3:**
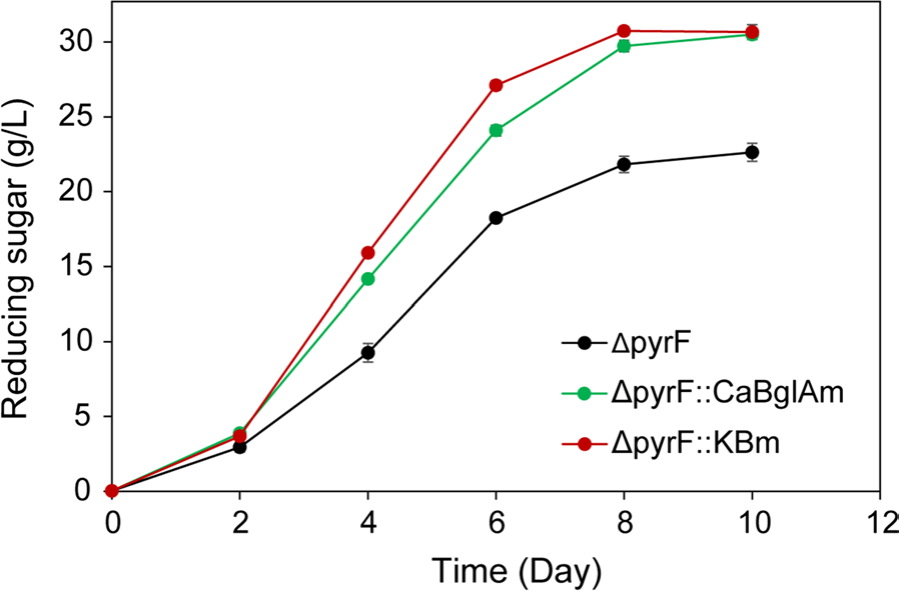
Cellulose saccharification of *C. thermocellum* strains with 40 g/L SPS as the substrate. The SPS saccharification was performed in GS-2 medium with an inoculum size of 1%. The amount of produced reducing sugar was determined by DNS method during the saccharification process. Three independent experiments were performed for every strain to calculate the average values and standard errors

With 40 g/L SPS as the cellulosic substrate, *∆pyrF*::KBm produced 30.75 g/L reducing sugar determined by 3,5-dinitrosalicylic acid (DNS) method. High-performance liquid chromatography (HPLC) analysis revealed that the produced reducing sugar contained 22.9 g/L glucose and 7.0 g/L xylose, and no cellooligosaccharides and cellobiose were detected. Because SPS contains 65.3% cellulose (0.71 g/g in glucose equivalent) and 16.3% hemicellulose (0.18 g/g in xylose equivalent), the saccharification levels of cellulose and hemicellulose were calculated as 80.6% and 97.2%, respectively. The higher saccharification level of hemicellulose than cellulose was reasonable because *C. thermocellum* cannot use xylose as the carbon source but can assimilate glucose, cellobiose and other cellooligosaccharides for growth [37]. In comparison, Δ*pyrF*::*Ca*BglAm could produce over 30 g/L reducing sugar but required two more days and Δ*pyrF* only produced 22.6 g/L sugar after 10-day saccharification (Fig. 3). Thus, *∆pyrF*::KBm showed the highest saccharification efficiency among tested strains. However, it still took a long time (8 days) for *∆pyrF*::KBm including a 2-day lag phase which might be caused by the low inoculum size, and the degree of efficiency improvement was not as obvious as that in microcrystalline cellulose (MCC) saccharification. Furthermore, the GS-2 medium commonly used for *C. thermocellum* cultivation contains expensive ingredients that are feasible economically. Hence, it is necessary to optimize the process and the medium to enhance the solubilization efficiency and reduce the cultivation cost.

### Optimization of saccharification medium

GS-2 medium was commonly used for the cultivation of *C. thermocellum* and other clostridial strains [38]. It contains cellobiose or cellulose as the carbon source, urea as the inorganic nitrogen source, cysteine hydrochloride as sulfur supply [39], yeast extract as a supply of both nitrogen and necessary trace elements, and other phosphates and salts. For saccharification, pretreated biomass is used instead of cellobiose or cellulose to reduce the cost of carbon source. However, the sulfur and nitrogen supplies are still at a high cost.

**Fig. 4:**
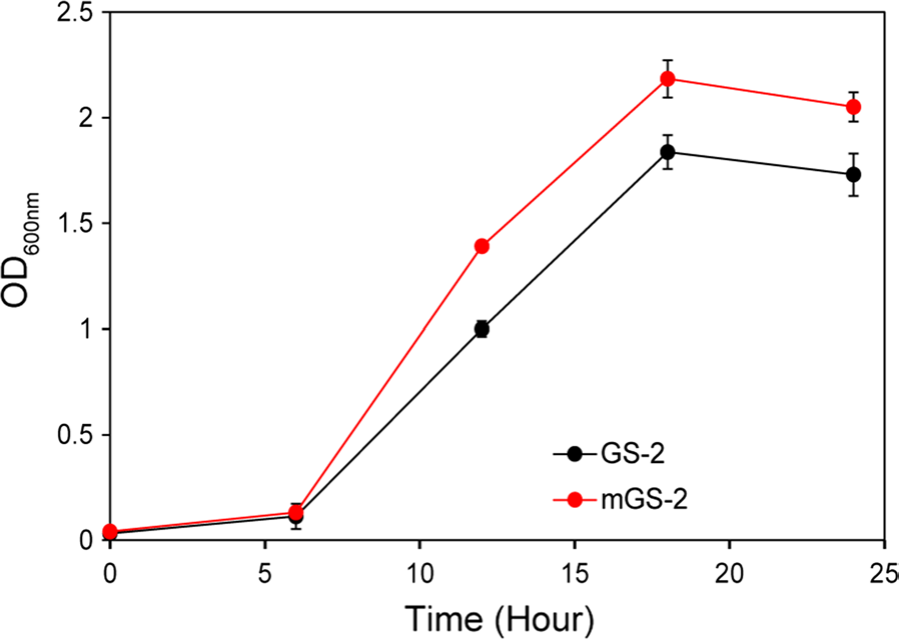
Growth curves of *C. thermocellum* strains. Cells were grown on GS-2 medium or modified GS-2 medium (mGS-2) containing 2 g/L sodium sulfide and 8 g/L corn steep liquor to substitute cysteine hydrochloride and yeast extract, respectively. 5 g/L cellobiose was used as the carbon source. The optical density at 600 nm was monitored to determine the cell growth. Three independent experiments were performed for every strain to calculate the average values and standard errors

We investigated the growth curves of cells cultivated in various modified media to determine the influence on cell growth. The media were derived from GS-2 containing cellobiose as the carbon source and substitutive ingredients. The results showed that cells growing in media containing 2 g/L sodium sulfide instead of 1 g/L cysteine hydrochloride or medium with 8 g/L corn steep liquor instead of yeast extract had similar growth patterns with those cultivated in GS-2 medium as shown in Additional file 3, indicating that sodium sulfide could be used as an alternative sulfur supply and corn steep liquor was used to replace yeast extract. The cell biomass obtained from the modified GS-2 (mGS-2) medium containing 2 g/L sodium sulfide and 8 g/L corn steep liquor was about 1.2-fold of that from GS-2 medium (Fig. 4).

*∆pyrF*::KBm was then used for saccharification using 100 g/L Avicel as the substrate and GS-2 or mGS-2 as the cultivation medium. As shown in Fig. 2, the sugar production rate obtained using mGS-2 maintained stably in Phase I but was greatly enhanced in Phase II from 1.90 to 3.26 g/L/day. Over 70 g/L reducing sugar was produced in 14 days using mGS-2 medium, which was 4 days earlier than that using GS-2 medium (Fig. 2). Additionally, the nitrogen and sulfur supplies in mGS-2 were cheap substitutes that only cost 0.23 and 0.087 US cents per liter, respectively. In contrast, the cost of yeast extract and cysteine hydrochloride is about 2.8 and 3.2 US cents per liter for the GS-2 medium, respectively. Thus, the cost of mGS-2 is much lower than that of GS-2. This result suggested that mGS-2 was a cost-effective medium for *C. thermocellum* cultivation to obtain more cell biomass for higher saccharification efficiency. Although *∆pyrF*::KBm can grow in both GS-2 and mGS-2 media without extra addition of uracil, stimulated cell growth was observed by adding uracil. This indicated that the complementation of the *pyrF* gene in the biocatalysts would be necessary before industrial application to further enhance the cell growth without increasing the medium cost. The mGS-2 medium was used for further saccharification analysis in this study.

### Optimization of biocatalyst cultivation and inoculation for SPS saccharification

**Fig. 5:**
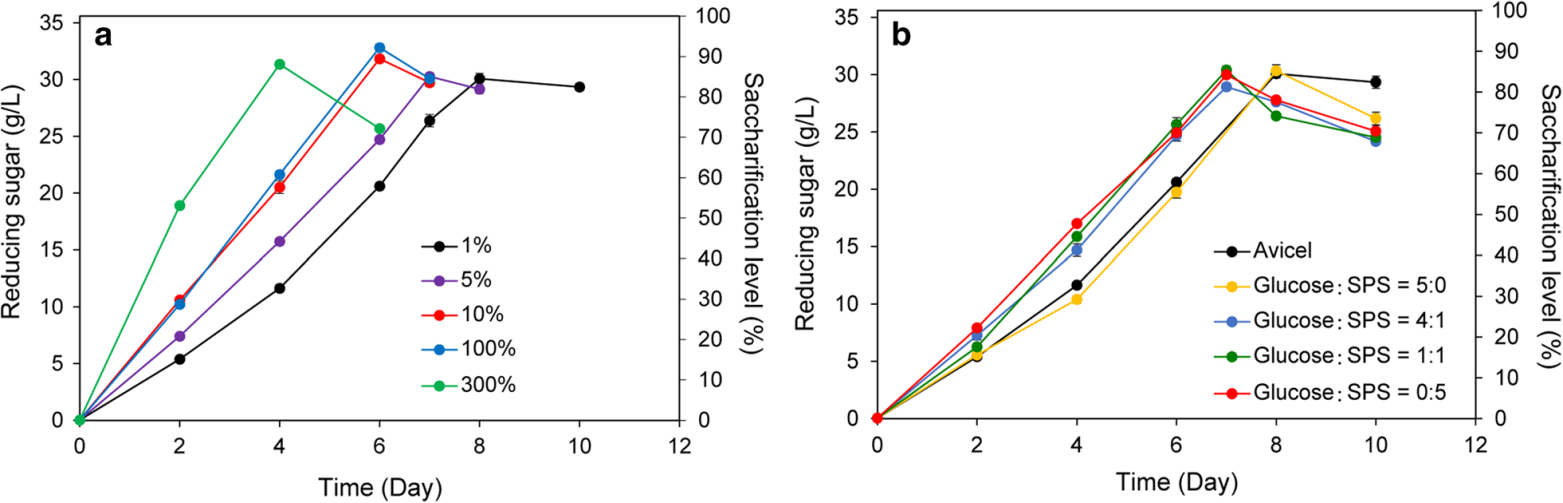
Influence of inoculum size (**a**) and cultivation (**b**) on saccharification of *C. thermocellum* strain *∆pyrF*::KBm. 40 g/L SPS was used as the substrate. **a** The inoculum size varied from 1% to 300%. **b** *∆pyrF*::KBm was cultivated with a mixture of glucose and SPS with a ratio of 5:0, 1:4, 1:1 or 0:5 as the carbon source. Avicel was used as a control. The saccharification level was calculated by dividing the initial polysaccharides in the substrate (~ 0.89 g/g) with the amount of the obtained reducing sugar, as determined by the DNS method. The inoculum size was 1%. Three independent experiments were performed to calculate the average values and standard errors

1% inoculum size may cause a lag phase of the saccharification process (Fig. 3). To optimize the inoculum size of SPS saccharification, the *∆pyrF*::KBm cells grown on Avicel until mid-log phase were used as the inoculum with a size of 1–300%, and stimulated saccharification efficiency was observed along with the increased inoculum size (Fig. 5a). With 5% inoculation, no apparent lag phase was observed and 30 g/L of reducing sugar was produced in 7 days instead of 8 days. With 10% inoculation, the saccharification process was further shortened by another 1 day to obtain a saccharification level of about 90%. Interestingly, no further enhancement of the saccharification process was observed when the inoculum size was increased from 10 to 100%. But the saccharification level could reach 88% in 4 days with 300% inoculation. In light of the operational feasibility and cost, 5 or 10% of inoculum size was considered to be conducive to eliminating the lag phase and promoting the saccharification process.

For the industrial purpose, the biocatalyst cultivation should not be performed using cellobiose or Avicel as the sole carbon source because of the high cost. *C. thermocellum* can also grow on glucose, a cheaper carbon source and one of the main products of cellulosic substrate saccharification, but with a long adaption phase [37]. SPS can be used as the carbon source for inoculum growth but the cells may attach to the substrate residues and cannot be easily separated for inoculation. Thus, to reduce cultivation cost under the premise of not reducing saccharification efficiency, glucose and SPS were tested as the carbon source for inoculum cultivation. Cells grown on cellobiose were inoculated in media with a mixture of glucose and SPS with a ratio of 5:0, 1:4, 1:1 or 0:5 as the carbon source and subcultured for two times for adaption and were cultivated till the mid-log phase. Avicel was used as the positive control. As shown in Fig. 5b, similar saccharification patterns were detected with cells grown on glucose or Avicel as the inoculum, and when SPS was used to grow the cells, with or without glucose and independent of the supplementation ratios, the saccharification process was stimulated and shortened by 1 day to reach the saccharification level of ~ 85%. This result suggested that SPS could be used as the sole carbon source for the cultivation of the whole-cell biocatalyst without adding high-price substrates.

### Influence of hemicellulases on SPS saccharification process

**Fig. 6:**
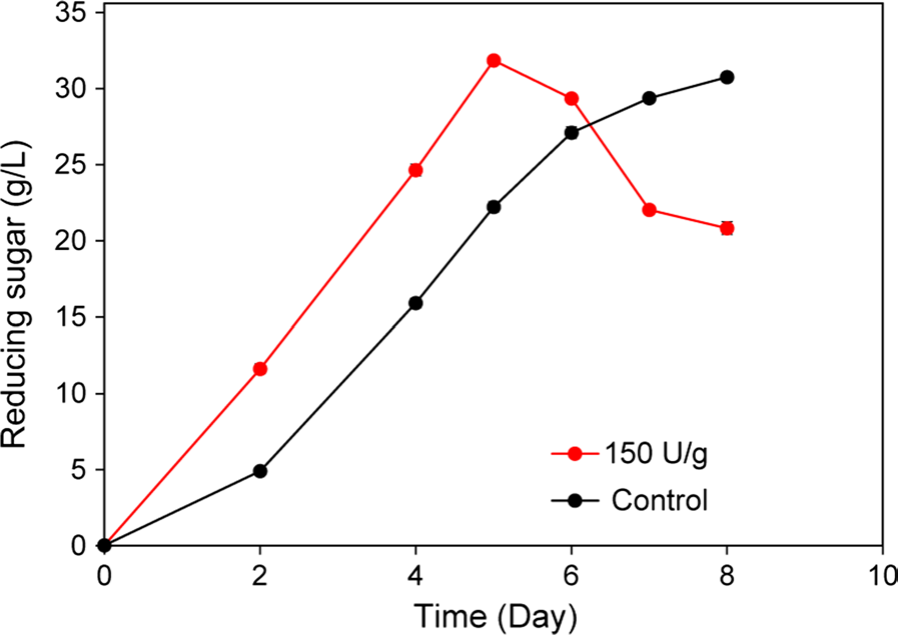
Supplementation of hemicellulase enhanced SPS saccharification. 40 g/L SPS was used as the substrate. The saccharification was performed with (150 U/g) or without (control) the addition of commercial hemicellulase cocktail. The saccharification level was calculated by dividing the initial polysaccharides in the substrate (~ 0.89 g/g) with the amount of the obtained reducing sugar as determined by the DNS method. The inoculum size was 1%. Three independent experiments were performed to determine the average values and standard errors

Although *C. thermocellum* produces several cellulosomal hemicellulases, it cannot grow on hemicellulose-derived sugars. It is presumed that the main role the hemicellulases play is to expose the cellulose fibers and make them more accessible to hydrolysis [40], and the hemicellulase activity of the cellulosome may not be sufficient in terms of the saccharification of pretreated biomass substrate. Thus, we added commercial hemicellulases into the saccharification system to enhance the hemicellulase activity. The result showed that the saccharification process was greatly shortened from 8 days to 5 days with the addition of 150 U/g hemicellulase cocktail, mainly due to the elimination of the 2-day lag phase (Fig. 6). This result demonstrated that high hemicellulose degradation activity was essential for increasing the initial hydrolysis rate. Thus, cellulosomal hemicellulases of the current biocatalyst could be further enhanced by overexpressing endogenous enzymes or introducing heterologous enzymes.

### Influence of substrate load on SPS saccharification process

**Fig. 7:**
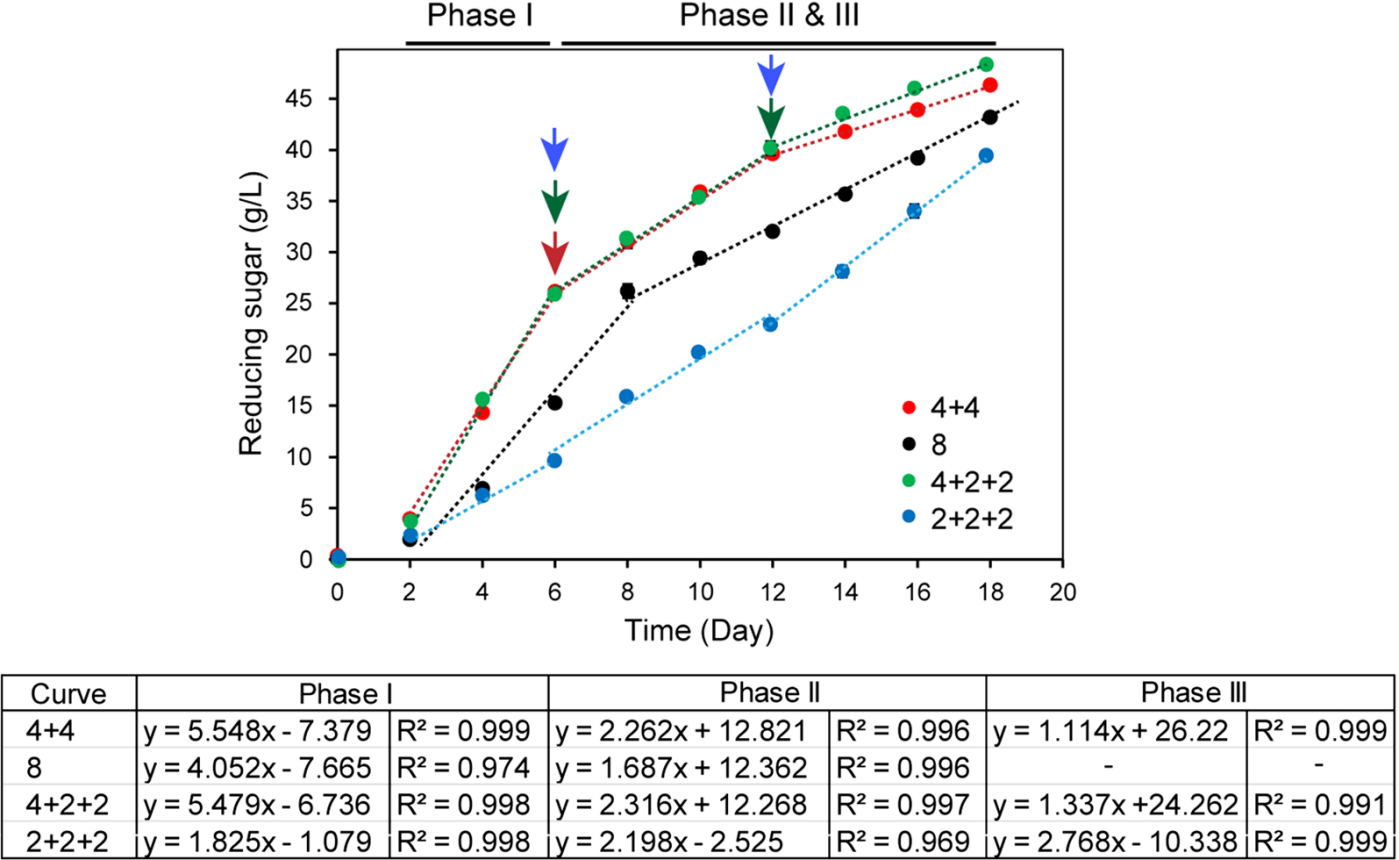
Influence of substrate load on SPS saccharification with or without substrate feeding. “4 + 4” with 40 g/L initial substrate and 40 g/L SPS was supplemented at 6th day; “8”, 80 g/L SPS was supplemented to initiate a batch saccharification process; “4 + 2 + 2” with 40 g/L or 20 g/L initial substrate and 20 g/L SPS were subsequently added at the 6th and 12th day; “2 + 2 + 2”, with initial substrate and 20 g/L SPS were added at the 6th and 12th day. The inoculum size was 1%. The saccharification process was divided into two or three phases based on the time points of substrate feeding (indicated by arrows with corresponding colors). The slopes and *R*^*2*^ values of the linear trend lines were calculated for all phases, and are shown in the table. The slope values stand for the sugar production rates (g/L/day). Three independent experiments were performed to determine the average values and standard errors

As mentioned above, over 30 g/L reducing sugar could be produced from 40 g/L SPS substrate using the newly developed biocatalyst *∆pyrF*::KBm, resulting in a saccharification level of around 90% (Fig. 3). To determine whether the saccharification efficiency would be influenced by substrate load, up to 80 g/L SPS was used for saccharification through batch and fed-batch substrate supplementation (Fig. 7). In the first 2 days, less than 5 g/L reducing sugars was produced with the initial SPS substrate load of either 20, 40 or 80 g/L. After the 2-day lag phase, the sugar production rates varied along with the substrate load significantly.

With 40 g/L initial substrate load, the production rate was around 5.5 g/L/day, but the value reduced to 4.0 and 1.8 g/L/day with 80 g/L and 20 g/L initial substrate load, respectively (Fig. 7). The results indicated that higher (80 g/L) or lower (20 g/L) initial substrate load was not beneficial for saccharification under the experimental conditions because low substrate load might cause insufficient carbon source for cellulosome production and, therefore, resulted in the decreased hydrolysis efficiency, and the horizontal shaking mode used in this study might result in inefficient mass transfer in the system when high substrate load was used, and further led to a declined sugar production. Thus, 40 g/L was the optimal initial substrate load to enhance saccharification efficiency under the experimental conditions.

At the 6th and 12th day, 20 g/L SPS was supplemented in the reactions with 40 g/L or 20 g/L initial substrate subsequently for fed-batch saccharification, termed “4 + 2 + 2” and “2 + 2 + 2”, respectively (Fig. 7). To reach a total substrate load of 80 g/L, 40 g/L SPS was supplemented at the 6th day in the system with 40 g/L initial substrate, termed “4 + 4” fed-batch process. For “4 + 4” and “8” processes which had the same total substrate load, decreased sugar production rates of 2.262 and 1.687 g/L/day were detected after 6 or 8 days’ saccharification, respectively. Because the initial sugar production rate of “4 + 4” process was higher than the 80 g/L SPS saccharification process, a higher amount of reducing sugars was produced in the fed-batch process (Fig. 7). This indicated that fed-batch, instead of batch saccharification, should be used to obtain a high amount of reducing sugars. For “4 + 2 + 2”, the sugar production rate was reduced to about 2.3 g/L/day and 1.3 g/L/day after the first and second substrate feeding, respectively. In contrast, the sugar production rate of the “2 + 2 + 2” process slightly increased from 1.8 g/L/day to 2.2 g/L/day and 2.8 g/L/day after the first and second substrate feeding (Fig. 7). However, the sugar production rate of the “2 + 2 + 2” process generally maintained at a low level, and it took 18 days to produce about 39.5 g/L reducing sugars. In comparison, a similar amount of reducing sugars was produced in “4 + 2 + 2” process at the 12th day before the second feeding. It is noteworthy that “4 + 2 + 2” and “4 + 4” showed similar saccharification patterns. This suggested that the substrate load affected saccharification efficiency at the beginning but had a slight influence in the later process.

### Consolidated bio-saccharification of SPS under optimal conditions

**Fig. 8:**
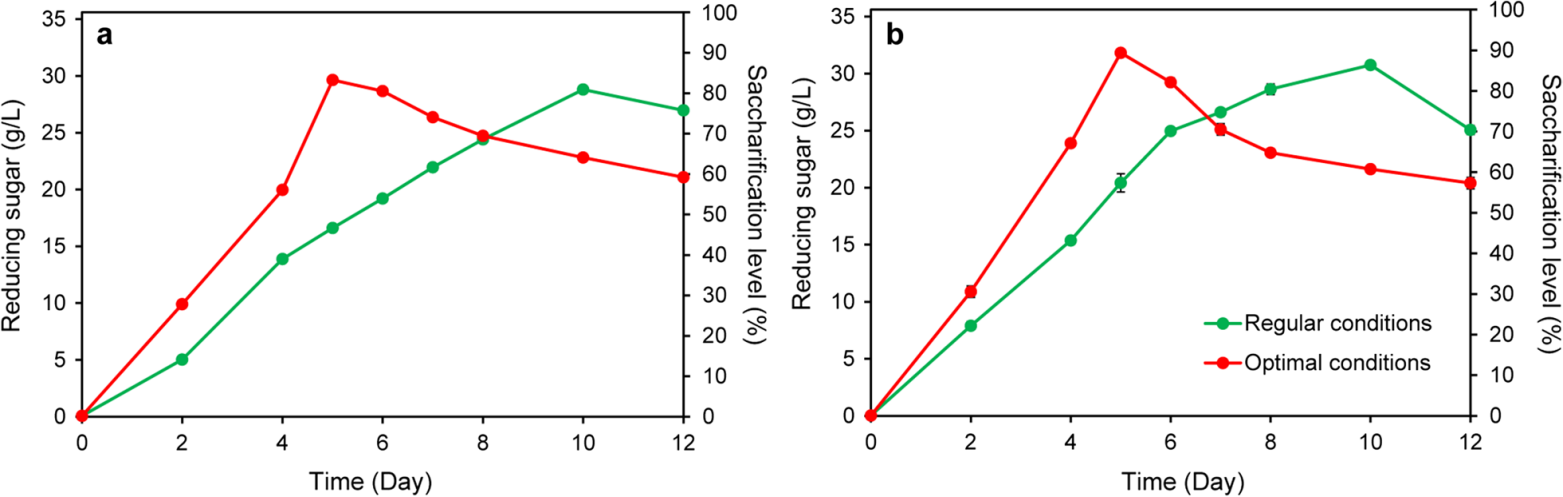
SPS saccharification under optimal or regular conditions without modification in both 100-mL anaerobic bottles (**a**) and a 10-L anaerobic fermenter (**b**). Regular conditions, ∆*pyrF*::*Ca*BglA as the biocatalyst, inoculum size of 1%, GS-2 medium. Optimal conditions, ∆*pyrF*::KBm as the biocatalyst, inoculum size of 5%, mGS-2 medium, 150 U/g hemicellulase. 40 g/L SPS was supplemented as the substrate. The saccharification level was calculated by dividing the initial polysaccharides in the substrate (~ 0.89 g/g) with the amount of the obtained reducing sugar, which was determined by the DNS method. Two (10 L reaction) or three (100 mL reaction) parallels were prepared for each experiment

Consolidated bio-saccharification of 40 g/L SPS was performed under optimal conditions (∆*pyrF*::KBm as the biocatalyst, inoculum size of 5%, mGS-2 medium, 150 U/g hemicellulase) or the regular conditions without modification (∆*pyrF*::*Ca*BglA, inoculum size of 1%, GS-2 medium) in both 100-mL anaerobic bottles (Fig. 8a) and a 10-L anaerobic fermenter (Fig. 8b). Additionally, we observed enhanced saccharification process at 60 °C compared to 55 °C, which was reasonable because the optimal growth temperature of *C. thermocellum* is 60 °C [41]. Under optimal conditions, over 30 g/L reducing sugar was produced in 5 days and the saccharification process was shortened by 50% compared to that under regular conditions (Fig. 8). The saccharification level was 89.3% and 0.795 g/g reducing sugar was produced from SPS. Hence, the saccharification efficiency was significantly stimulated using the newly constructed biocatalyst under the improved conditions. In this way, the consolidated bio-saccharification strategy could be applied for highly efficient lignocellulose solubilization.

## Conclusion

As a consolidated bioprocess, CBS employs cellulolytic microorganisms as a biocatalyst for lignocellulose saccharification and produces fermentable sugars as a product for various downstream use; thus, having the advantages of low cost and flexible application. This work provided a new CBS biocatalyst to improve the saccharification efficiency, and the saccharification process was thoroughly investigated and optimized. The CBS process was shortened by 50%, and the production cost was also reduced using inexpensive ingredients for biocatalyst cultivation. Thus, current CBS is feasible for cost-effective lignocellulose solubilization, which is the key step in the industrial utilization of lignocellulosic biomass.

## Methods

### Bacterial strains and cultivation

The bacterial strains used in this study are listed in Table 1. *Escherichia coli* strains were cultivated aerobically at 37 °C in Luria–Bertani (LB) liquid medium with shaking at 200 rpm or on solid LB plate with 1.5% agar. *C. thermocellum* strains were grown anaerobically at 55 or 60 °C in GS-2 or MJ medium with 5 g/L glucose, cellobiose, Avicel (PH-101, Sigma) or dry weight sulfite-pretreated wheat straw as the carbon source. A modified medium (mGS-2) was developed based on GS-2 by replacing yeast extract with corn steep liquor and cysteine hydrochloride with sodium sulfite. Corn steep liquor with 50% dry weight was purchased from AngelYeast Co., Ltd. 30 μg/mL chloramphenicol, 50 μg/mL kanamycin and 3 μg/mL thiamphenicol (Tm) were supplemented to the medium when necessary. 10 μg/mL 5-fluoro-2-deoxyuridine (FUDR) or 500 μg/mL 5-fluoroorotic acid (FOA) dissolved in dimethyl sulfoxide was added for screening.

**Table 1:** Bacterial strains and plasmids used in this study

Strains/plasmids	Relevant characteristic	Sources
Strains		
*E. coli*		
DH5α	*f80dlacZΔM15, Δ(lacZYA*-*argF)U169, deoR, recA1, endA1, hsdR17(rk*−*, mk*+*), phoA, supE44, l*−*, thi*-*1, gyrA96, relA1*	Transgen
BL21(DE3)	*ompT gal dcm lon hsdSB(rB*−*mB*−*) l (DE3 [lacI lacUV5*-*T7 gene 1 ind1 sam7 nin5])*	Transgen
*C. thermocellum*		
DSM1313	Wild-type stain	DSMZ
*ΔpyrF*	Derived from DSM1313, with deleted *pyrF* gene	[28]
*∆pyrF*::*Ca*BglA	Derived from *ΔpyrF*, producing a fusion protein of Cel48S and *Ca*BglA	[28]
*∆pyrF*::*Ca*BglAm	Derived from *ΔpyrF*, producing a fusion protein of Cel48S and *Ca*BglAm	[28]
*ΔpyrF*::KBm	Derived from *ΔpyrF*, producing a fusion protein of Cel9K and *Ca*BglA	This work
Plasmids		
pHK-HR-*Ca*BglA	Seamless genome editing plasmid for markerless knock-in of *caBglA* in the chromosome of DSM 1313	[28]
pHK-HR-KBm	Derived from pHK-HR-*Ca*BglA, with different homology arms	This work

### Plasmid construction

The plasmid pHK-HR-KBm was constructed based on previously reported pHK-HR-*Ca*BglAm [28] for the seamless integration of the codon-optimized β-glucosidase (BGL)-encoding gene *caBglAm* (GenBank Accession number: KY418041) in the chromosome of *C. thermocellum* strain *∆pyrF* [28]. The plasmid pHK-HR-*Ca*BglAm contains the endogenous selection marker *pyrF* (Clo1313_1266) driven by its native promoter, the selection marker *tdk* (Teth514_0091) from *Thermoanaerobacter* sp. X514 expressed under the control of an endogenous glyceraldehyde-3-phosphate dehydrogenase (*gapDH*) promoter, and three regions of homology, HR-up, HR-short, and HR-down [28]. Homology arms K-up, K-short, and K-down were amplified from the genome DNA of *C. thermocellum* DSM1313 using the primer set up-1/2, short-1/2, and down-1/2 (Table 2) to substitute HR-up, HR-short, and HR-down of pHK-HR-*Ca*BglAm using restriction sites *Xba*I/*Sal*I, *Xho*I/*Eag*I, and *Mlu*I/*Bam*HI, respectively (Additional file 1). K-short had the same sequence with the 3′ region of K-up. Because K-up has the homologous sequence with 3′-region of the catalyzing module (GH9) of the gene *cel9K* (Clo1313_1809) and K-down has the downstream genomic sequence of GH9 containing the dockerin module (DocI), *Ca*BglAm would locate in the middle of GH9 and DocI of Cel9K (Additional file 1).

**Table 2:** Primers used in this study

Primers	Sequences (5′–3′)^a^
Up-1	GTCTCTAGACATTGGCATTCTTCTATCACAAG (*Xba*I)
Up-2	GTCGTCGACTCCTCCTCCTGGCGGTGTTATT (*Sal*I)
Short-1	GTCCTCGAGGGACTGGATCAGTCCTATG (*Xho*I)
Short-2	GTCCGGCCGTCCTCCTCCTGGCGGTGTTATT (*Eag*I)
Down-1	GTCGCGCGCGGAGGAGGAGTAGACCCAGAAGAACCGGAG (*Mlu*I)
Down-2	GTCGGATCCACTACAACGCCTGCAGCATTC (*Bam*HI)

^a^Restriction sites are underlined and indicated in following parentheses

### Electrotransformation and screening of *C. thermocellum*

The constructed pHK-HR-KBm was transformed to *E. coli* BL21(DE3) to remove Dcm methylation [42], and transformed to *C. thermocellum* DSM1313 mutant Δ*pyrF* as previously described [43]. The cells were then recovered and screened on solid GS-2 medium containing Tm. The transformants containing the plasmid pHK-HR-KBm were then successively screened with FUDR-supplemented MJ medium lacking uracil and FOA-supplemented GS-2 medium to screen for mutant strains through two rounds of recombination as previously described [28]. Colony PCR and sequencing were performed using the primer up-1/down-2 to verify the chromosomal integration of the gene *caBglAm*. If a fragment with a band size of about 4.7 Kb was found, the target mutant strain Δ*pyrF*::KBm was obtained, which would express a fused protein of Cel9K and *Ca*BglAm containing three functional modules (GH9-*Ca*BglAm-DocI).

### Protein preparation and analyses

*Clostridium thermocellum* strains were cultivated in GS-2 medium with 5 g/L Avicel as the sole carbon source at 55 °C till the late lag phase. The supernatants were then obtained by centrifugation and used to prepare the extracellular proteins by condensing and cellulosomal proteins according to a modified cellulose affinity procedure as previously described [43]. The heterologous expression and purification of *Ca*BglA were performed using the previously reported strain *E. coli* BL21(DE3)::pET28aNS-*Ca*BglA and protocol [28]. All protein samples were stored at − 80 °C for further analysis. The protein concentrations were determined using the Bradford method [44] immediately before further analyses. Sodium dodecyl sulfate-polyacrylamide gel electrophoresis (SDS-PAGE) was performed to check protein purity and composition as previously described using protein standards ranging from 10 to 245 kDa (New England BioLabs) [33].

### Enzyme assay

The BGL activity was determined against *p*-nitrophenyl-β-d-glucopyranoside (pNPG) according to the previously reported method [28]. One unit of enzyme activity was defined as the amount of enzyme required to produce 1 μmol of *p*-nitrophenol (pNP) per min under certain conditions.

### Cellulosic substrate saccharification

*Clostridium thermocellum* strains were initially cultivated with 5 g/L Avicel as the sole carbon source until the mid-log phase unless otherwise stated. 1%, 5%, 10%, 100% and 300% (v/v) of the cells were inoculated into GS-2 or mGS-2 medium to initiate the saccharification process with MCC or SPS as cellulosic substrates. For 100% and 300% inoculation, cells from 100 to 300 mL culture were concentrated, re-suspended, and re-inoculated into 100 mL fresh mGS-2 medium anaerobically as the biocatalyst to initiate the saccharification process. The starting material of SPS was pretreated in a cooking digester (VRD-42SD-A, Beijing Pulp and Paper Research Institute, Beijing, China) with the ammonium sulfite dosage of 20 wt% at 160 °C for 2 h. Upon completion of pretreatment, the stock was taken out and transferred to a nylon bag (300 meshes) to separate the solid stock and spent liquor. After that, the treated stock was washed to neutrality with de-ionized water and stored at 4 °C for further analyses and saccharification. The chemical compositions of the raw and pretreated samples were determined on the basis of the National Renewable Energy Laboratory (NREL) procedure with the standard two steps of sulfuric acid hydrolysis [45]. The acid and saccharification filtrates were tested by HPLC equipped with a Bio-Rad Aminex HPX-87P column at 55 °C. The ultra-pure water was used as a mobile phrase with a flow rate of 0.55 mL/min. The lignin, extractives and sugar contents of wheat straw were also measured following the same NREL method [45]. The anaerobic bottles were horizontally shaken in a 55 °C incubator at 170 rpm unless otherwise stated. When MCC was used as the substrate, 100 g/L Avicel was added in the reaction system and the saccharification process lasts for 16–20 days. For SPS saccharification, 40–80 g/L SPS, containing 32.7–65.4 g/L polysaccharides (cellulose and hemicellulose) in equivalence, was supplemented in the reaction system and the saccharification process lasts for about 6–12 days. According to the experimental requirements, the saccharification reactions were performed at 55 or 60 °C, 170 rpm with volumes of 100 mL to 10 L. 1.5 mL cultures were sampled every 2 days and centrifuged at 13,000 rpm for 5 min. The supernatants were used to determine the production of reduced sugar by the 3,5-dinitrosalicylic acid (DNS) method and other metabolites using high-performance liquid chromatography (HPLC) as previously described [46]. The saccharification efficiency of the strain was defined by determining the saccharification level and sugar production rate. The former one was calculated by dividing the amount of initial polysaccharides (cellulose and hemicellulose in glucose and xylose equivalent, respectively) in the substrate with that of the obtained reducing sugar, and could be used to characterize the sugar yield as a percentage of the theoretical value. The latter was determined by calculating the amount of sugar produced per day per liter. Two or three independent parallels were set up for each strain.

## Additional files

**Additional file 1: Figure S1.** Schematic illustrating the plasmid pHK-HR-KBm and its usage in the knock-in of gene *caBglA* in the chromosome of *C. thermocellum* ∆*pyrF.* The plasmid was constructed based on the previously reported pHK-HR-*Ca*BglAm [28] by replacing the homologous arms with the regions of homology according to *cel9K* gene location in the genome of *C. thermocellum* DSM1313. The primer binding sites are indicated by arrows. The restriction sites and the length of the homologous arms are shown. The upstream arm K-up contains the sequence homologous with 3′-region of GH9 module of *cel9K* gene (yellow square). The downstream arm K-down contains the sequence homologous with DocI of *cel9K* gene (blue square). K-short has the same sequence with the 3′ region of K-up (dashed square). The *caBglA* gene (pink square) should be inserted in the middle of GH9 and DocI of Cel9K. The strain screening contains two rounds of recombination as previously described [28]. The obtained recombinant strain ∆*pyrF*::KBm would produce a fused protein containing 3 functional modules (GH9-CaBglAm-DocI) under the control of the endogenous Cel9K promoter.

**Additional file 2: Figure S2.** Avicel saccharification by ∆*pyrF*::KBm with supplementation of 0, 15 or 50 U/g cellulose of purified *Ca*BglA protein. The concentration of produced reducing sugar was determined by DNS method.

**Additional file 3: Figure S3.** Growth curves of *C. thermocellum* DSM1313 grown on various media with 5 g/L cellobiose as the carbon source. a, cells were grown with 1 g/L cysteine hydrochloride or 2 g/L sodium sulfide as sulfur supply. b. cells were grown on GS-2 medium containing 6 g/L yeast extract or modified media with 2 to 8 g/L corn steep liquor instead. The optical density at 600 nm was monitored to determine the cell growth. Three independent experiments were performed to calculate the average values and standard errors.

## Abbreviations

BGL: beta-glucosidases
CBP: consolidated bioprocessing
CBS: consolidated bio-saccharification
DNS: 3,5-dinitrosalicylic acid
FOA: 5-fluoroorotic acid
FUDR: 5-fluoro-2-deoxyuridine
gapDH: glyceraldehyde-3-phosphate dehydrogenase
HPLC: high-performance liquid chromatography
LB: Luria–Bertani
MCC: microcrystalline cellulose
pNP: *p*-nitrophenol
pNPG: *p*-nitrophenyl-β-d-glucopyranoside
SDS-PAGE: sodium dodecyl sulfate-polyacrylamide gel electrophoresis
SHF: separate enzymatic hydrolysis and fermentation
SPS: sulfite-pretreated wheat straw
SSF: simultaneous saccharification and fermentation
Tm: thiamphenicol
