# Supplementary figures and images for: Construction of consolidated bio-saccharification biocatalyst and process optimization for highly efficient lignocellulose solubilization
Source: Biotechnol Biofuels. 2019 Feb 18;12:35. doi: 10.1186/s13068-019-1374-2 (PMC6378752; doi:10.1186/s13068-019-1374-2)

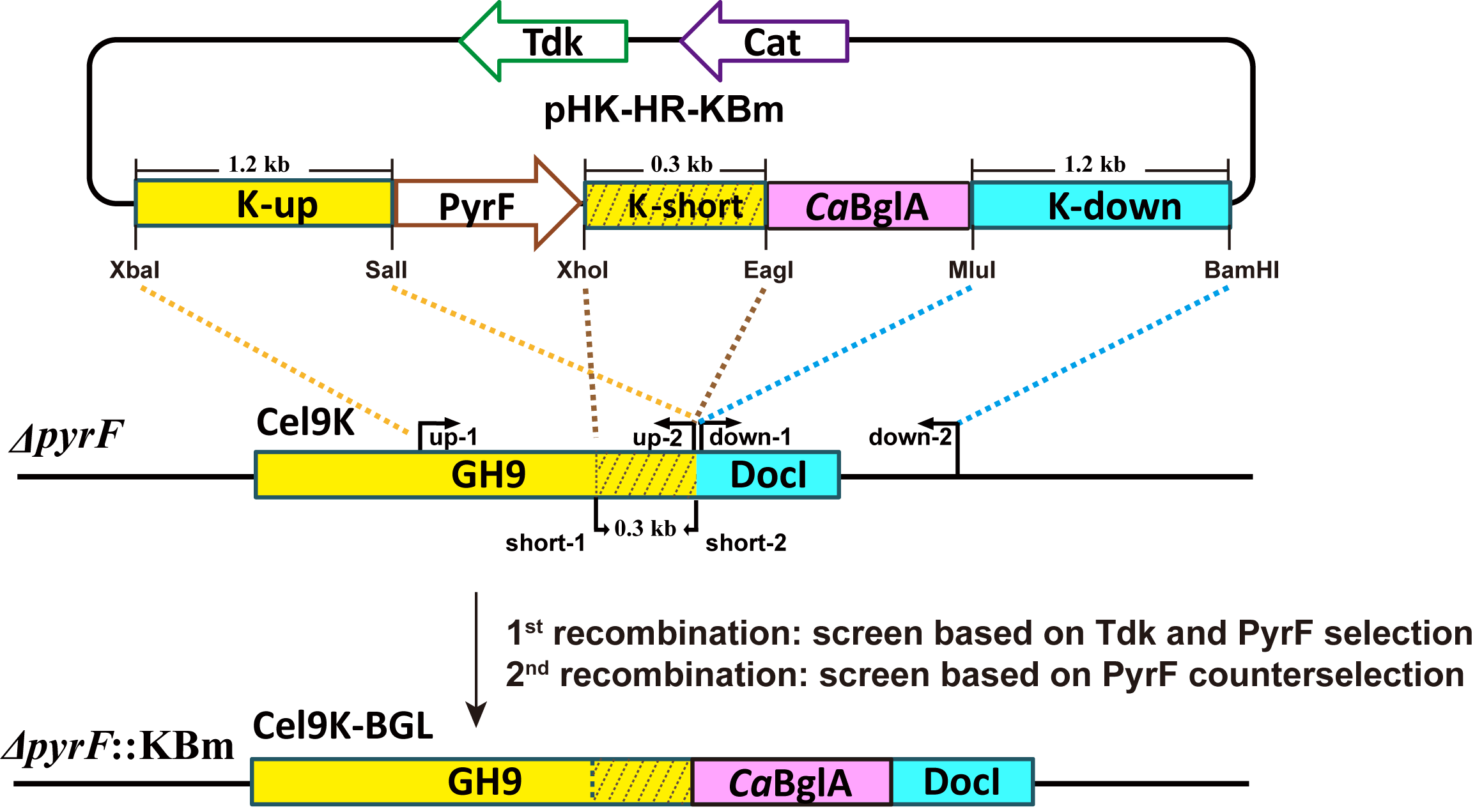

Supplement: Supplementary file 1 — Additional file 1: Figure S1. Schematic illustrating the plasmid pHK-HR-KBm and its usage in the knock-in of gene caBglA in the chromosome of C. thermocellum ∆pyrF. The plasmid was constructed based on the previously reported pHK-HR-CaBglAm [28] by replacing the homologous arms with the regions of homology according to cel9K gene location in the genome of C. thermocellum DSM1313. The primer binding sites are indicated by arrows. The restriction sites and the length of the homologous arms are shown. The upstream arm K-up contains the sequence homologous with 3′-region of GH9 module of cel9K gene (yellow square). The downstream arm K-down contains the sequence homologous with DocI of cel9K gene (blue square). K-short has the same sequence with the 3′ region of K-up (dashed square). The caBglA gene (pink square) should be inserted in the middle of GH9 and DocI of Cel9K. The strain screening contains two rounds of recombination as previously described [28]. The obtained recombinant strain ∆pyrF::KBm would produce a fused protein containing 3 functional modules (GH9-CaBglAm-DocI) under the control of the endogenous Cel9K promoter. [file 13068_2019_1374_MOESM1_ESM.tif]

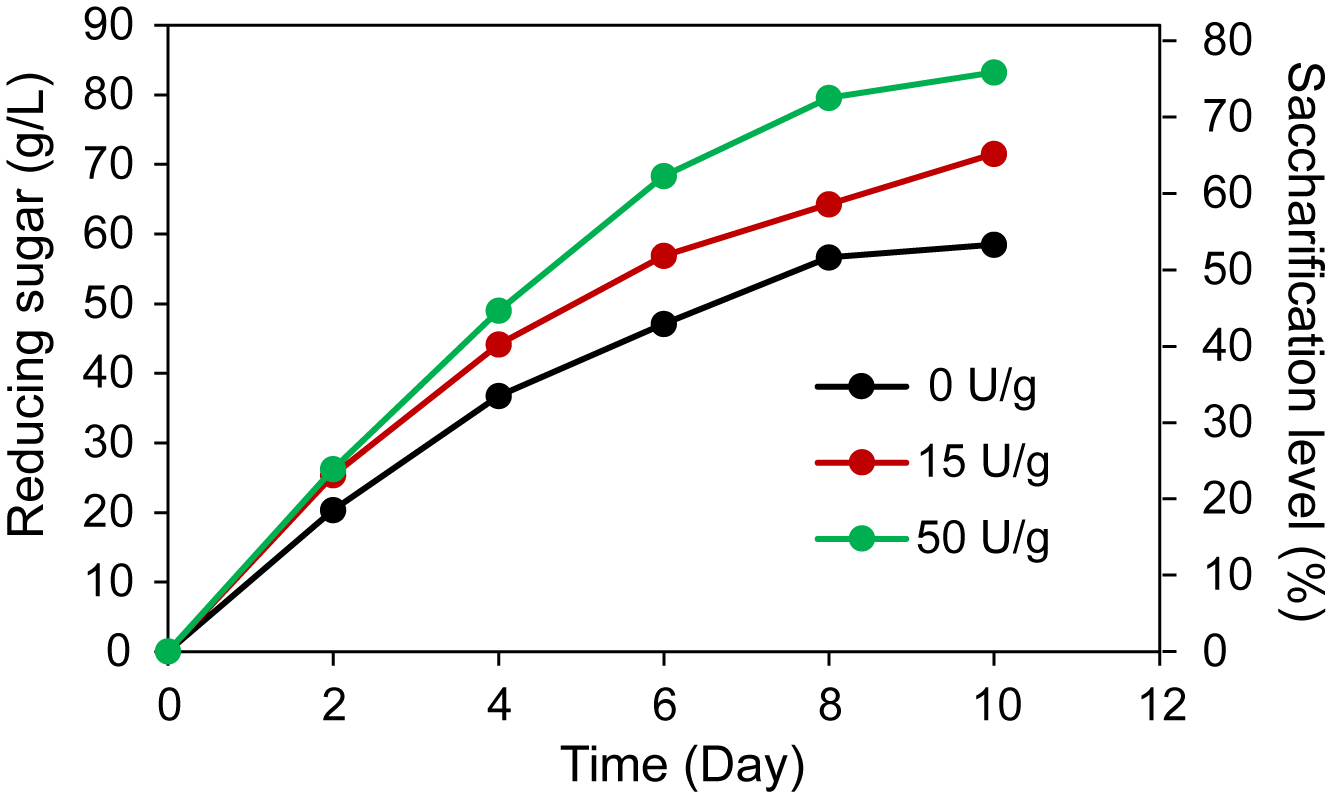

Supplement: Supplementary file 2 — Additional file 2: Figure S2. Avicel saccharification by ∆pyrF::KBm with supplementation of 0, 15 or 50 U/g cellulose of purified CaBglA protein. The concentration of produced reducing sugar was determined by DNS method. [file 13068_2019_1374_MOESM2_ESM.tif]

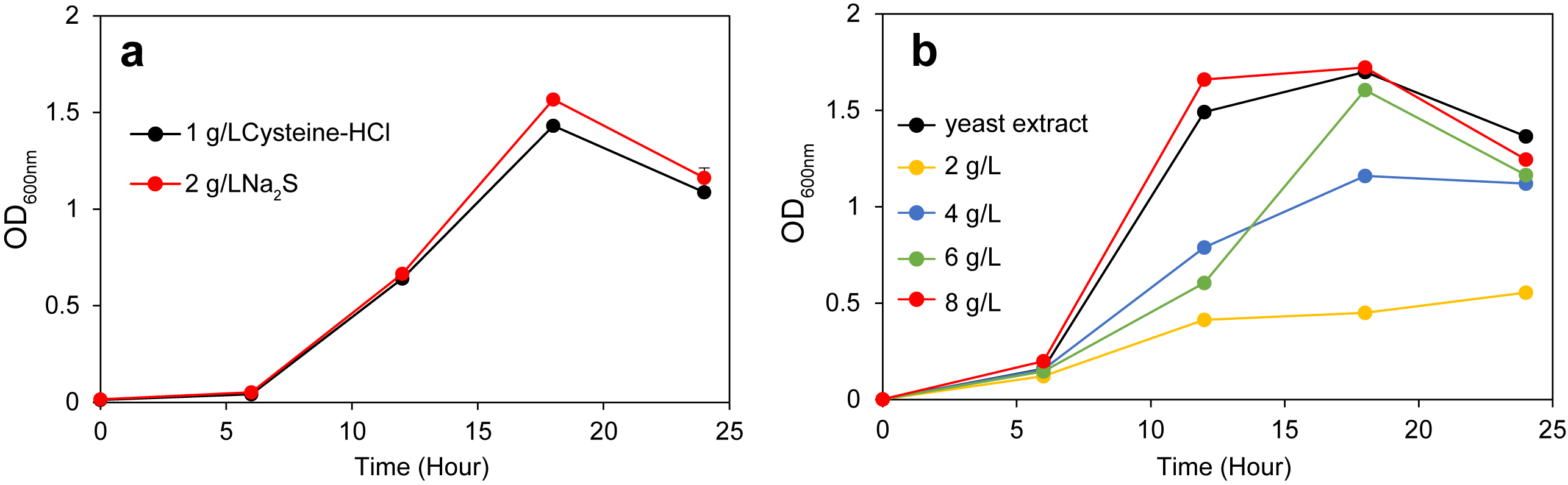

Supplement: Supplementary file 3 — Additional file 3: Figure S3. Growth curves of C. thermocellum DSM1313 grown on various media with 5 g/L cellobiose as the carbon source. a, cells were grown with 1 g/L cysteine hydrochloride or 2 g/L sodium sulfide as sulfur supply. b. cells were grown on GS-2 medium containing 6 g/L yeast extract or modified media with 2 to 8 g/L corn steep liquor instead. The optical density at 600 nm was monitored to determine the cell growth. Three independent experiments were performed to calculate the average values and standard errors. [file 13068_2019_1374_MOESM3_ESM.tif]
